# Positive dysphotopsia after intrascleral intraocular lens fixation: a case report

**DOI:** 10.1186/s12886-022-02474-z

**Published:** 2022-06-11

**Authors:** Tsuyoshi Mito, Honoka Kawakami, Toru Ikoma, Yuki Ukai, Mizuho Tsuchiya, Eri Kubo, Hiroshi Sasaki

**Affiliations:** 1 Department of Ophthalmology, Kanazawa Medical University, 1-1 Daigaku, Uchinada, Kahoku-Gun, Ishikawa, 920 -0293 Kanzawa, Japan; 2 Tsuchiya Eyeclinic, Ishikawa, Japan

**Keywords:** Positive dysphotopsia, Intrascleral intraocular lens fixation, Peripheral iridectomy, Intraocular lens edge

## Abstract

**Background:**

Positive dysphotopsia is a symptom caused by the reflection of incident light through the pupil at the inner surface of the intraocular lens (IOL) edge after cataract surgery and is perceived as an abnormal arcuate or radiating photopic image at night or indoors with a light source. Although positive dysphotopsia is one of the most important symptoms that affect patients after cataract surgery, it is still not well known even among ophthalmologists. Positive dysphotopsia as the cause of patient complaint following intraocular surgery other than cataract surgery has not been identified.

**Case Presentation:**

A 52-year-old man underwent IOL extraction and intrascleral IOL fixation for bilateral IOL subluxation at another hospital. The right eye had good subjective visibility, but the patient noticed symptoms of light sources appearing divided into multiple lights indoors after surgery in the left eye. Because the cause of the symptoms could not be identified, the patient visited our department. At the time of his first visit, the corrected visual acuity in both eyes was good, and ocular findings in eye position, motility, intraocular pressure, and fundus were within normal limits. The elongated holes of peripheral iridectomy (PI) created during previous intrascleral IOL fixation were observed to be approximately 2 mm in length on the nasal side in both eyes. The PI hole in the right eye was covered by the optics of the IOL, whereas the edge of the IOL overlapped the center of the PI hole in the left eye. Accordingly, we concluded that the abnormal photopic image in the left eye was caused by positive dysphotopsia, in which light passing through the PI hole was reflected by the edge of the IOL. We attempted surgical closure of the PI hole, resulting in the complete disappearance of positive dysphotopsia.

**Conclusions:**

A PI hole created during intrascleral IOL fixation may cause postoperative positive dysphotopsia depending on the position of the IOL edge. Thus, surgeons should be aware of the importance of the size and location of the PI hole when creating it during surgery.

## Background

Dysphotopsia is a photopic phenomenon that affects patients who undergo cataract surgery, causing symptoms such as abnormal halos of light and shadows; the former is called positive dysphotopsia (PD) and the latter negative dysphotopsia [1]. PD became progressively better known in the late 1990s and is recognized as an abnormal arcuate or radiating photopic image at night or indoors with a light source. According to previous reports, most symptoms are related to the nature of the inserted intraocular lens (IOL) [2]. Although the incidence rate of PD at 1 year after cataract surgery is 0.2–2.2% [1, 3], there might be some cases in which a patient with abnormal photopic phenomena is under observation without identifying the cause. Moreover, there have been no reports of PD in intraocular surgeries other than cataract surgery to date.

Here, we report a case of postoperative PD in a patient who underwent intrascleral IOL fixation for IOL subluxation.

## Case presentation

**Fig. 1:**
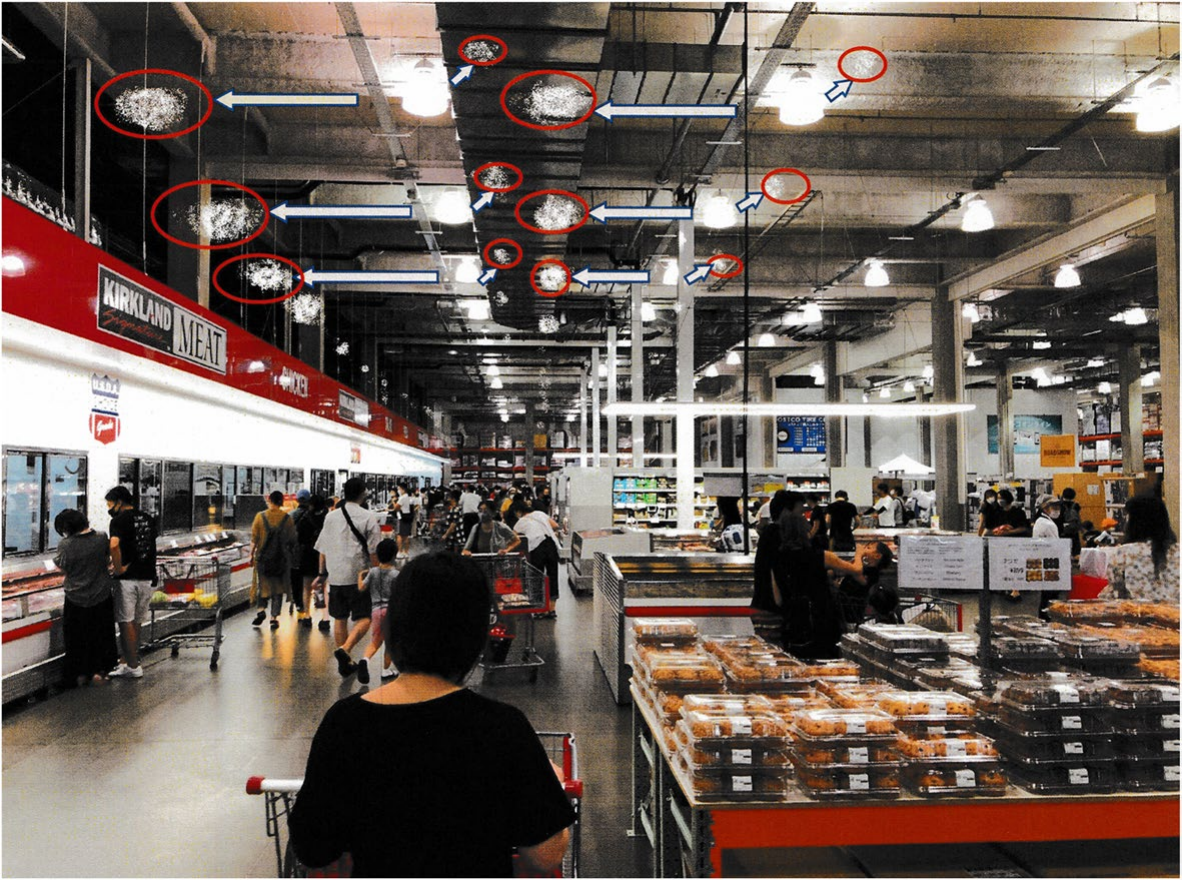
Image graphics prepared by the patient according to his complaints. He explained that the light sources indoors were divided into multiple lights (red circle part) when viewed only by the left eye. The image depicted here is our own property

A 52-year-old man underwent IOL extraction and intrascleral IOL fixation in the right eye in 2018 and the left eye in 2020 because of IOL subluxation in both eyes. The patient’s postoperative subjective visibility was good in the right eye; however, the patient noticed symptoms of light sources appearing to be divided into multiple lights indoors in the left eye immediately after surgery, which interfered with his daily life. Figure 1 represents the image of the patient’s symptoms experienced in a shopping center, showing ceiling lights as multiple lights. Because the cause of the symptoms could not be identified at the hospital where the surgery was performed, the patient visited our department for further investigation.

**Fig. 2:**
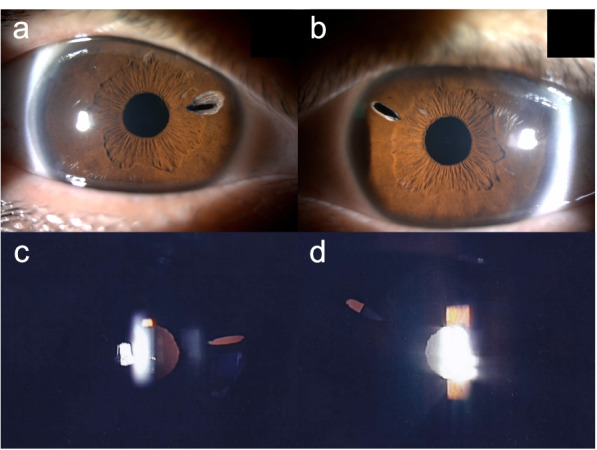
Slit-lamp photomicrographs at the initial diagnosis in the right eye (**a, c**) and left eye (**b, d**). The PI holes with approximately the same size and position in both eyes were observed. In the retroillumination image, the right eye is covered by the IOL optics (**c**), whereas the IOL edge was observed in the center of the PI hole in the left eye (**d**)

**Fig. 3:**
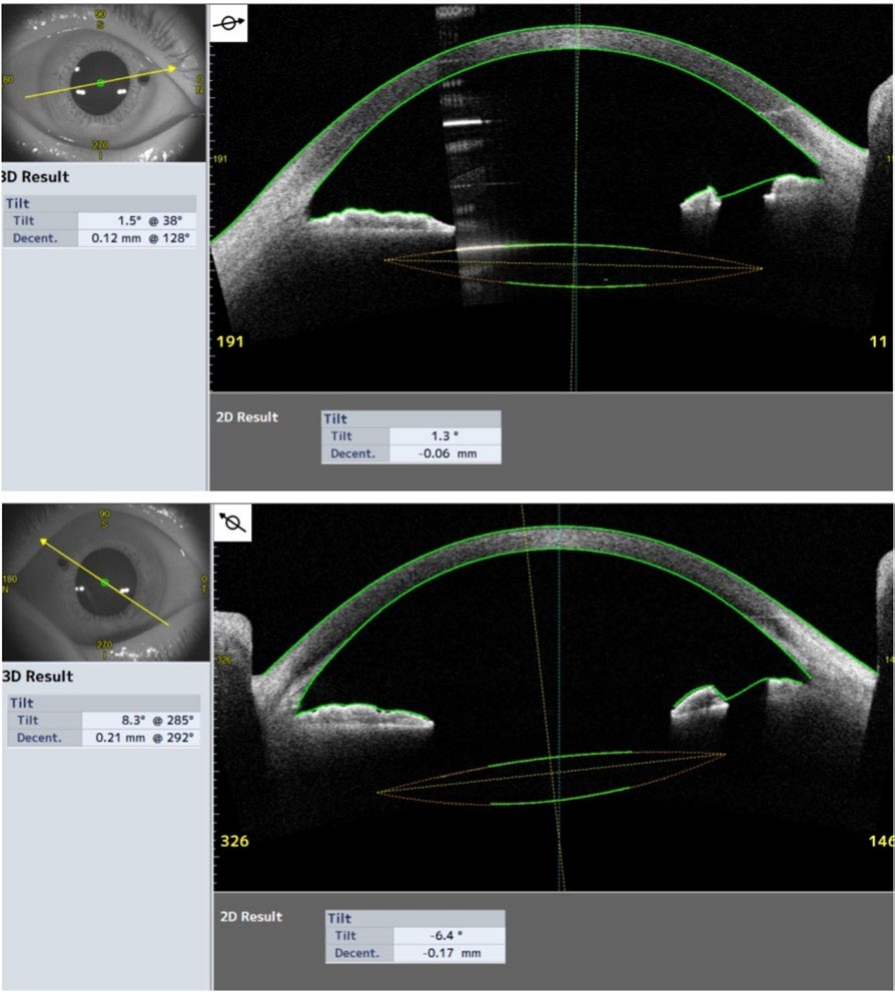
Anterior ocular segment optical coherence tomography findings. A slight tilt was observed in the left eye compared to the right eye. Under dark conditions, the IOL optical surface directly below the PI hole was observed in the right eye, whereas the PI hole overlapped the IOL edge in the left eye

At his first visit to our department, his corrected visual acuity (VA) was 20/16 and 20/20 in the right and left eyes, respectively. The ocular findings in eye position, ocular motility, intraocular pressure, and fundus were within normal limits. The pupils were circular and of the same size on both sides; however, the elongated holes of peripheral iridectomy (PI) created during the past intrascleral IOL fixation were observed to be approximately 2 mm in length on the nasal or supra-nasal sides in both eyes (Fig. 2a-b). The PI hole in the right eye was covered by the optics of the IOL, whereas the edge of the IOL overlapped the center of the PI hole in the left eye (Fig. 2c-d). The decentrations of the IOL fixed in the sclera were 0.12 mm and 0.21 mm, and the tilts of the IOL were 1.5° and 8.3° in the right and left eyes, respectively. The left eye had a slightly larger IOL tilt, and the edge of the IOL overlapped the PI hole in the left eye observed by anterior segment optical coherence tomography (CASIA2; Tomey Corporation, Nagoya, Japan) (Fig. 3). Thus, we concluded that the abnormal photopic phenomena in the left eye were caused by PD, in which light passing through the PI hole was reflected by the edge of the IOL and formed an ectopic image on the retina.

**Fig. 4:**
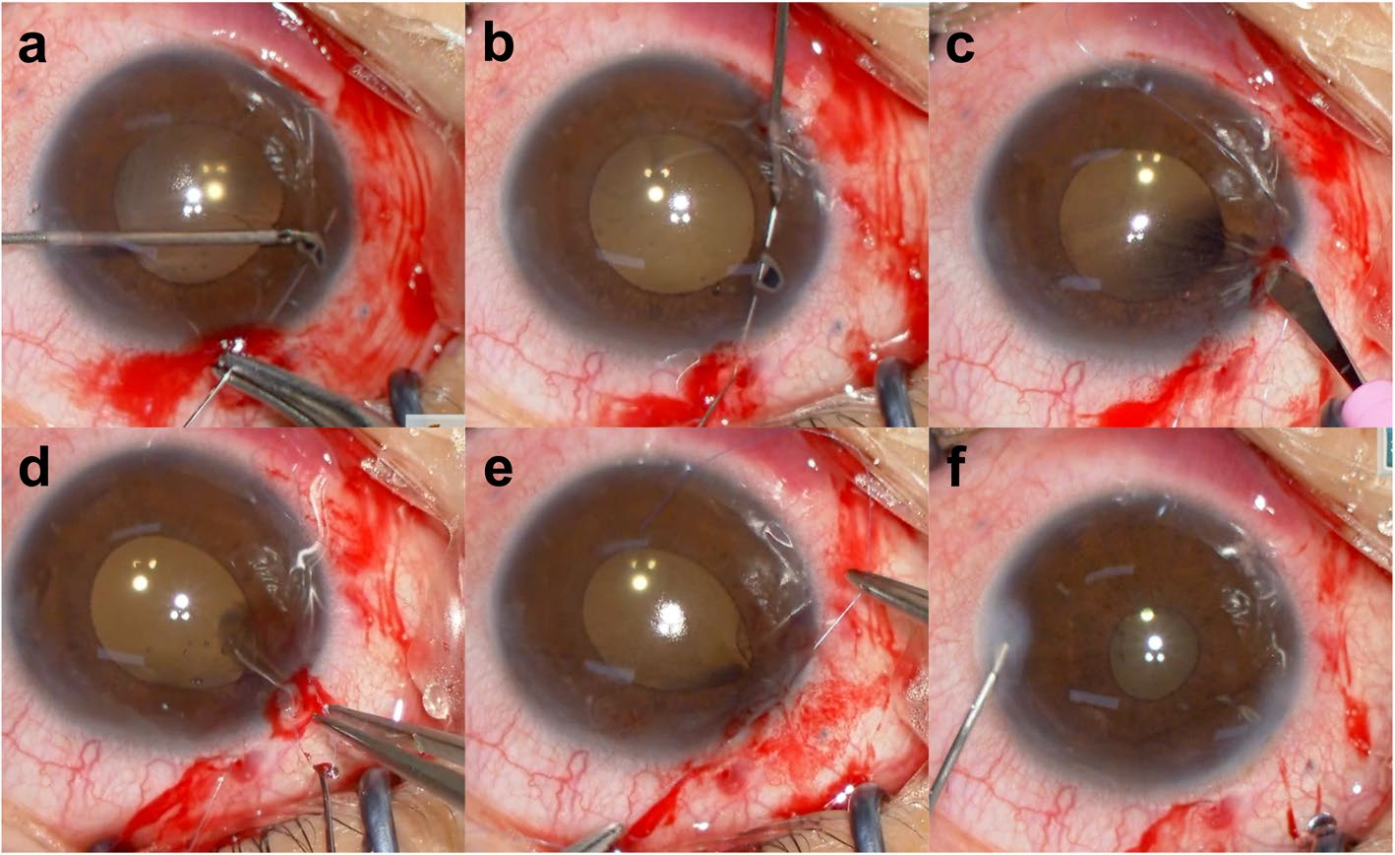
Video images during surgery. The center of the PI hole was pierced with a 10–0 polypropylene suture (**a**). The needle was pulled out of the eye using a 27-gauge needle (**b**). A corneal incision of approximately 1.5 mm wide was created directly above the PI hole (**c**). The 10–0 polypropylene suture was pulled out of the eye through the corneal incision from the anterior chamber (**d**). The suture was ligated out of the eye (**e**). The ligature was returned into the eye (**f**)

**Fig. 5:**
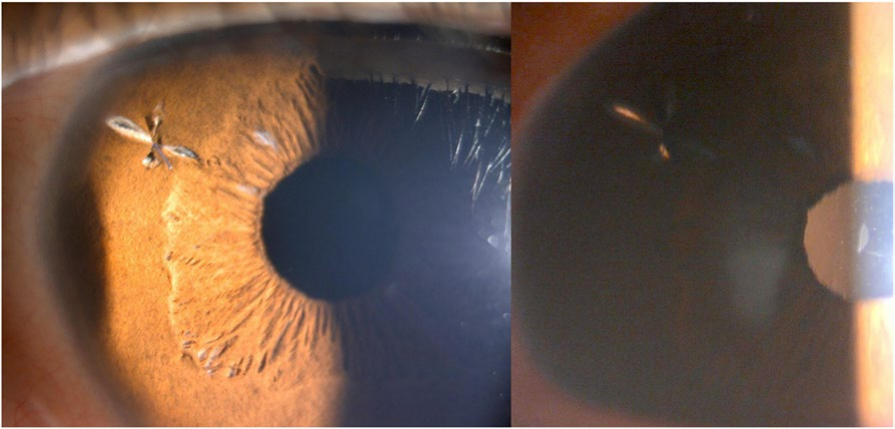
Postoperative slit-lamp photomicrographs. The PI hole was closed, and the IOL edge is not observed in the retroillumination image

We attempted surgical closure of the PI hole using the McCannel method [4]. Briefly, the center of the PI hole was pierced with a 10–0 polypropylene suture, and the needle was pulled out of the eye. Then, a main incision of approximately 1.5 mm wide was created directly above the PI hole, the suture through the PI hole was pulled out of the eye from the anterior chamber, ligated, and trimmed to return the ligature to the eye (Fig. 4a-f), thereby closing the PI hole (Fig. 5). The subjective symptoms of PD completely disappeared, the postoperative corrected VA was unchanged from the preoperative corrected VA, higher order aberrations measured with wavefront analyzer KR-1 W (Topcon, Tokyo, Japan) showed no abnormalities before and after surgery, and no iris capture of IOL or intraocular pressure elevation has been observed to date.

## Discussion and conclusions

In general, PD occurs in patients who have IOL inserted in cataract surgery and is caused by light rays from the outside passing through the pupil to the IOL, with some light reflecting off the inner surface of the IOL edge and forming an image on the retina [5], causing strong complaints in some cases. This is the first report of PD following intrascleral IOL fixation, possibly caused by the reflection of light at the edge of the IOL through the PI hole created during intrascleral IOL fixation. PD disappeared completely after surgical closure of the PI hole, supporting this hypothesis.

In recent years, intrascleral IOL fixation and transscleral suture fixation of the IOL have increased due to the increased number of cases of ciliary zonule weakness or avulsion and postoperative decentration or dislocation of the IOL. One of the postoperative complications is iris capture of the IOL and IOP elevation associated with pupillary block [6]. Occasionally, a PI hole is created intraoperatively to avoid these postoperative complications, but there is no clear consensus on the size and position of the PI hole. In this case, PD did not develop in the right eye, even though the PI holes were of the same size and position on the nasal or supranasal sides in both eyes. The main cause of PD is suggested to be the overlap between the PI hole and the edge of the IOL. The PI hole did not overlap the edge of the IOL in the right eye, whereas the edge of the IOL was visible in the center of the PI hole in the left eye.

There are no reports of PD in IOL eyes after laser peripheral iridotomy (LI) or trabeculectomy surgery, even though they are similar to IOL eyes with iris defects, which is the onset factor of PD in this report. This may be because the LI hole rarely overlaps the edge of the IOL as the peripheral iris, where the distance between the iris and anterior lens subcapsule can be secured, is usually used as the irradiation position in cases of LI, and an extremely large LI hole is rarely created to avoid incident light from the LI hole. In contrast, PD has not been reported in IOL eyes after trabeculectomy surgery, which may cause larger iris defects, because glaucomatous visual field defects may make it difficult to recognize PD; the iris defect may be easily hidden by the upper eyelid because the bleb is inevitably located in the upper part of the eye. However, the mechanism of dysphotopsia may not be simple, as there are report of dysphotopsia being induced by the tear meniscus even when the LI hole is hidden by the eyelid [7]. As PD is still not well known among ophthalmologists, it may be overlooked; thus, it is necessary to accumulate cases to determine if any pseudophakic LI or trabeculectomy cases develop PD. If the IOL is completely covered with continuous curvilinear capsulorrhexis during cataract surgery in patients undergoing LI or trabeculectomy, the anterior lens subcapsule gradually becomes opaque, and light does not penetrate to the edge of the IOL. Accordingly, even if PD occurs, the symptoms may disappear spontaneously. In this respect, since the lens capsule covering the IOL is absent after intrascleral IOL fixation or transscleral suture fixation of the IOL, PD is likely to occur at the exposed edge of the IOL.

In this case, it was known preoperatively that covering the PI hole with his finger caused the PD to disappear, so it could have been treated conservatively by wearing contact lenses with an iris design; however, it is unclear how much improvement can be achieved. There is also a treatment method for keratopigmentation with black ink. However, the difficulty is that staining the cornea above the edge of the IOL requires staining the area near the center of the cornea. In addition, Jabbour et al*.* reported that corneal staining with black ink for dysphotopsia (e.g., glare and light scatter) after LI in phakic eyes weakens its long-term effects [8]. Therefore, surgical closure of the PI hole, as in this case, may be the most effective treatment.

We encountered a case of PD after intrascleral IOL fixation, which was caused by an overlap of the PI hole and the edge of the IOL. Surgeons who perform intrascleral IOL fixation or transscleral suture fixation of the IOL should be aware that creating an intraoperative PI hole may cause postoperative PD.

## Data Availability

All data generated or analyzed during this study are included in this published article.

## Abbreviations

IOL: Intraocular lens
LI: Laser peripheral iridotomy
PD: Positive dysphotopsia
PI: Peripheral iridectomy
VA: Visual acuity

## References

[1] Davison JA (2000). Positive and negative dysphotopsia in patients with acrylic intraocular lenses. J Cataract Refract Surg.

[2] Masket S, Fram NR (2021). Pseudophakic Dysphotopsia: Review of Incidence, Cause, and Treatment of Positive and Negative Dysphotopsia. Ophthalmology.

[3] Ellis MF (2001). Sharp-edged intraocular lens design as a cause of permanent glare. J Cataract Refract Surg.

[4] McCannel MA (1976). A retrievable suture idea for anterior uveal problems. Ophthalmic Surg.

[5] Holladay JT, Lang A, Portney V (1999). Analysis of edge glare phenomena in intraocular lens edge designs. J Cataract Refract Surg.

[6] Yamane S, Sato S, Maruyama-Inoue M, Kadonosono K (2017). Flanged Intrascleral Intraocular Lens Fixation with Double-Needle Technique. Ophthalmology.

[7] Vera V, Naqi A, Belovay GW, Varma DK, Ahmed II (2014). Dysphotopsia after temporal versus superior laser peripheral iridotomy: a prospective randomized paired eye trial. Am J Ophthalmol.

[8] Jabbour S, Choremis J, Boutin T, Brunette I, Mabon M, Talajic JC (2019). Poor Long-Term Outcomes of Keratopigmentation With Black Ink for the Treatment of Dysphotopsia Secondary to Laser Peripheral Iridotomies. Cornea.

